# Exploring the validity of estimating EQ-5D and SF-6D utility values from the health assessment questionnaire in patients with inflammatory arthritis

**DOI:** 10.1186/1477-7525-8-21

**Published:** 2010-02-11

**Authors:** Mark J Harrison, Mark Lunt, Suzanne MM Verstappen, Kath D Watson, Nick J Bansback, Deborah PM Symmons

**Affiliations:** 1 The arc Epidemiology Unit, The University of Manchester, Oxford Road, Manchester, M13 9PT, UK; 2 Centre for Health Evaluation and Outcome Sciences, St. Paul's Hospital, 570-24 1081 Burrard Street, Vancouver, V6Z 1Y6, Canada

## Abstract

**Background:**

Utility scores are used to estimate Quality Adjusted Life Years (QALYs), applied in determining the cost-effectiveness of health care interventions. In studies where no preference based measures are collected, indirect methods have been developed to estimate utilities from clinical instruments. The aim of this study was to evaluate a published method of estimating the EuroQol-5D (EQ-5D) and Short Form-6D (SF-6D) (preference based) utility scores from the Health Assessment Questionnaire (HAQ) in patients with inflammatory arthritis.

**Methods:**

Data were used from 3 cohorts of patients with: early inflammatory arthritis (<10 weeks duration); established (>5 years duration) stable rheumatoid arthritis (RA); and RA being treated with anti-TNF therapy. Patients completed the EQ-5D, SF-6D and HAQ at baseline and a follow-up assessment. EQ-5D and SF-6D scores were predicted from the HAQ using a published method. Differences between predicted and observed EQ-5D and SF-6D scores were assessed using the paired t-test and linear regression.

**Results:**

Predicted utility scores were generally higher than observed scores (range of differences: EQ-5D 0.01 - 0.06; SF-6D 0.05 - 0.10). Change between predicted values of the EQ-5D and SF-6D corresponded well with observed change in patients with established RA. Change in predicted SF-6D scores was, however, less than half of that in observed values (p < 0.001) in patients with more active disease. Predicted EQ-5D scores underestimated change in cohorts of patients with more active disease.

**Conclusion:**

Predicted utility scores overestimated baseline values but underestimated change. Predicting utility values from the HAQ will therefore likely underestimate the QALYs of interventions, particularly for patients with active disease. We recommend the inclusion of at least one preference based measure in future clinical studies.

## 

The assessment of the cost-effectiveness of health care interventions has become increasingly important as health care providers aim to select the treatments and interventions which maximise health gain from their scarce resources. Assessments based on quality-adjusted life years (QALYs) are used to compare the benefits of interventions across medical conditions. The calculation of QALYs involves weighting duration of life by a preference-based measure of the health-related quality of life (HRQol) experienced. Preference based measures are based on methods to value health states using simulated choices between alternative health states: an individual considers a transition from a defined health state to some alternative (usually preferable) health state which involves a sacrifice of something they value, for example life expectancy, or a risk of an unfavourable event such as death. The greater the sacrifice or risk accepted to make the transition, the lower the valuation of the defined health state [1]. Preference based measures provide a value (known as utility), on a scale ranging from 1 (equivalent to full health) to 0 (equivalent to death) with the potential in some measures for states considered 'worse than death.' The calculation of cost per QALY as a basis for assessing the cost-effectiveness of a treatment has been adopted by organisations evaluating and recommending treatments in many countries including the UK [2] and the USA[3]

Preference based measures such as the EuroQol-5D (EQ-5D) [4] and the Short Form-6D (SF-6D)[5] which is derived from the Short Form 36-Item Health Survey (SF-36)[6]) collect information about the health status of patients using self-administered questionnaires. The health status of the patient is then linked to a societal utility value, one aimed to be representative of the values of the population of a particular country, which is obtained via large valuation studies in the general population which attribute a utility value to each possible health state described by the questionnaire.

In rheumatology, most clinical studies incorporate the Health Assessment Questionnaire Disability Index (HAQ)[7], which is a condition-specific health status measure that focuses on functional disability, a single aspect of health. Condition-specific health status measures have limited use in economic evaluation because comparison across therapeutic areas becomes almost impossible. Since treatments for rheumatology have to 'compete' with treatments for other diseases, the comparison of cost-effectiveness using generic outcome measures is essential.

Despite their importance, many studies do not collect generic preference based utility measures. To overcome this limitation, methods of estimating the utility values of preference based measures from disease specific measures have been developed. In rheumatology, a model has recently been developed which maps the HAQ to the EQ-5D and SF-6D for the purpose of estimating the average utility of a cohort [8]. The use of mapping techniques has been described as second-best compared to primary collection of data [9], but remain one of the most practical solutions available when no utility measure has been collected. Since the inclusion of preference based measures increases the number of items in collected in a study, adding to patient burden, and are often seen as less important than clinical outcome measures, it might also be deemed necessary to use these mapping functions in future studies. In these circumstances, the performance of the mapping function in estimating utility values needs to be assessed and the likely impact of decisions based on these estimates considered. Data supporting the construct validity and responsiveness of the SF-6D derived from the HAQ [8] has been reported in patients with early aggressive RA[10]. However, to date there has been no evaluation of EQ-5D values predicted from the HAQ, and neither EQ-5D nor SF-6D scores predicted from the HAQ have to date been compared with actual measured values. The aim of this study was to evaluate the published method of estimating mean EQ-5D and SF-6D utility scores from the Health Assessment Questionnaire (HAQ), by comparing measured and predicted values in groups of patients with inflammatory arthritis with varying arthritis states and degrees of disease severity.

## Methods

### Patients and Setting

Data were taken from three cohorts of patients. The first was The Steroids in Very Early Arthritis (STIVEA) randomised controlled trial (RCT) of intramuscular steroid treatment versus placebo in patients with very early inflammatory arthritis (4-11 weeks duration). The trial follow-up finished in late 2007 [11]. At the time of this analysis, the STIVEA trial remained blinded. The trial analysis has since shown that although treatment with intramuscular steroids postponed the use of DMARDs and prevented 1 in 10 patients with very early IP from progressing to rheumatoid arthritis, there was no statistically significant difference between the two treatment arms in any of the secondary outcome measures (which included HAQ, the SF-36 and the EQ-5D) at 6-months nor 12 months of follow-up [11].

The second cohort comprised patients from the British Rheumatoid Outcome Study Group (BROSG) RCT of aggressive versus symptomatic control of inflammation in patients with established (>5 years duration) stable, symptomatic rheumatoid arthritis (RA) followed for three years. The BROSG trial was conducted between 1998 and 2001 [12]. The BROSG trial found no difference between treatment arms (aggressive versus symptomatic treatment aimed at suppressing inflammation) over a three year period. Thus, the dataset may be considered a cohort of patients with established RA whose RA deteriorated modestly over a three year period [6].

The third cohort was a sub-sample from the British Society for Rheumatology Biologics Register (BSRBR) of UK RA patients receiving anti-TNF therapy. The BSRBR was established in October 2001, and the methods of this study have been described in detail previously [13]. Briefly, the first 4000 RA patients starting each anti-TNFα therapy were required by The National Institute for Health and Clinical Excellence (NICE) to be registered with the BSRBR and followed up for information on drug use, disease activity and adverse events. Routine data collection includes the HAQ and SF-36. As part of the current study, from 1^st ^August 2006 to 31^st ^December 2007, patients were also asked to complete the EQ-5D at baseline and the 6 month assessment.

The data from these three cohorts reflect a wide range of arthritis states/severity found in routine practice. Baseline data for all cohorts included age, sex and disease duration. Patients also completed the EQ-5D[4], and the SF-36[6] which is used to calculate the SF-6D utility measure[5]. The HAQ (adjusted for aids/devices and help from others), a patient global assessment, the 28 tender and swollen joint counts and the erythrocyte sedimentation rate (ESR) were collected, and the Disease Activity Score (DAS-28)[14] was calculated (Table 1).

**Table 1:** Summary of outcome measures used in this study

	Type of measure	Range of scores	
		Worst	Best
EQ-5D	Preference based utility measure/HRQoL	-0.59	1.00
SF-6D	Preference based utility measure/HRQoL	0.30	1.00
HAQ^†^	Functional disability	3	0
DAS28^†^	Disease activity	10	0
28 Tender joint count^†^	Physician assessment of tenderness in 28 joints	28	0
28 Swollen joint count^†^	Physician assessment of swelling in 28 joints	28	0
ESR (mm/hr)^†^	Laboratory test of inflammatory marker/acute phase reactant	*	0

Abbreviations: DAS28 = Disease Activity Score based on 28 swollen and tender joint counts, EQ-5D = EuroQol-5D, ESR = Erythrocyte Sedimentation Rate, HAQ = Health Assessment Questionnaire, HRQoL = Health-Related Quality of Life, SF-6D = Short Form-6D

* Higher values indicate inflammation

### Statistical Methods

Baseline characteristics were summarised and compared between cohorts using the Kruskal-Wallis test for continuous variables and the Chi-square test for categorical variables.

Estimated EQ-5D and SF-6D scores were calculated from the HAQ, using the most successful of the mapping methods described in the article by Bansback *et al*. [8]. The methods were developed cross-sectional data from a cohort of 439 patients with a clinical diagnosis of RA from two locations (308 participating in a study in Vancouver, Canada (mean (SD) age 61.4 (13.7) years, 78% female, mean (SD) disease duration 14.0 (12.6) years), and 131 participating in a study in Maidstone, UK (mean (SD) age 56.0 (13.7) years). The mean (SD) HAQ score of the patients used by Bansback *et al*. was 1.15 (0.78) and scores ranged from 0 to 3. EQ-5D and SF-6D scores were estimated from items from the HAQ using linear regression models estimated by generalised estimating equation algorithms. Full regression equations for estimating the EQ-5D and SF-6D from the HAQ are reported in the original study by Bansback, *et al*. [8] and an example of how to use the algorithms is available online http://www.pharmacoeconomics.ubc.ca/download.html.

In this study, we estimated the EQ-5D using model 5 described by Bansback, *et al*., which was based on the individual items of the HAQ, and treating each as a categorical variable[8]. We estimated the SF-6D using model 2 from the paper which used the 8 HAQ domain scores, treated as a continuous variable[8]. These models were reported to have the lowest mean square error and the best predictive value of the five methods.

In order to investigate the relationship between the HAQ and the EQ-5D and SF-6D as a basis for mapping, we tested associations between the HAQ, EQ-5D and SF-6D at baseline and for change over time using Spearman's rank because the HAQ and EQ-5D are non-normally distributed. The mean predicted and observed EQ-5D and SF-6D scores were compared for each cohort at baseline and in terms of the change between baseline and the final follow-up. The mean differences between predicted and observed values were calculated and presented with 95% confidence intervals and a 95% reference range, Differences between the mean observed and predicted scores for a group were tested using the paired t-test. The correlations of observed and predicted values for each measure were assessed as an indicator of the performance of the prediction model, using the R^2 ^statistic from a linear regression.

## Results

### Cross-sectional analysis

265 patients recruited to STIVEA, 466 to BROSG, and 866 patients from the BSRBR received a baseline EQ-5D and SF-36 questionnaire. 1472 patients completed and returned all the baseline questionnaires and were included in this analysis; 224 (85%) of the STIVEA cohort, 453 (97%) of the BROSG cohort, and 795 (92%) of the BSRBR patients.

There were significant differences in demographic and clinical characteristics between the three groups (Table 2). Patients from the BROSG study were older (median 62 years) than those from STIVEA (median 59 years) and BSRBR (median 59 years) studies, and had lower DAS28 scores (median: BROSG 4.0 vs. STIVEA 5.5 and BSRBR 6.0) and lower median tender (median: BROSG 3 vs. STIVEA 9 and BSRBR 12) and swollen joint counts (median: BROSG 3 vs. STIVEA 8 and BSRBR 7). There was a trend of increasing HAQ score with increasing disease duration (i.e. STIVEA>BROSG>BSRBR), but only the difference between patients in the STIVEA (median 1.3) and BSRBR (median (IQR) 1.8) studies was statistically significant (p < 0.001). There were proportionally more women in the BSRBR study (76%) than the BROSG (68%) or STIVEA (72%) studies (p = 0.003). Baseline correlations of HAQ and EQ-5D scores ranged from r = 0.63 (BROSG & BSRBR) to r = 0.69 (STIVEA), and between HAQ and SF-6D from r = 0.58 (BROSG) to r = 0.68 (STIVEA & BSRBR) (results not provided in tables).

**Table 2:** Baseline characteristics of patients from the three cohorts, ranked by median HAQ score

	STIVEA	BROSG	BSRBR	
	n = 224	n = 453	n = 795	p-value*
Age (years)	59 (44, 66)	62 (53, 69)	59 (51, 67)	<0.001
Disease duration (years)	0.16 (0.12, 0.19)	11 (7, 16)	9 (3, 18)	<0.001
Female gender, n(%)	160 (72%)	308 (68%)	604 (76%)	0.009†
HAQ	1.3(0.6, 1.6)	1.5 (0.9, 2.0)	1.8 (1.1, 2.1)	<0.001
DAS28	5.5 (4.8, 6.4)	4.0 (3.2, 4.9)	6.0 (5.1, 6.8)	<0.001
28-Tender joint count	9 (5, 15)	3 (1, 8)	12 (6, 19)	<0.001
28-Swollen joint count	8 (5, 12)	3 (1, 6)	7 (4, 12)	<0.001

Values are median (IQR) unless otherwise stated. * Kruskal-Wallis; † Chi-square

Abbreviations: BROSG = British Rheumatoid Outcome Study Group, BSRBR = British Society for Rheumatology Biologics Register, DAS28 = Disease Activity Score based on 28 swollen and tender joint counts, HAQ = Health Assessment Questionnaire, STIVEA = Steroids In Very Early Arthritis,

Overall, the predicted values of the SF-6D (R^2 ^0.34 - 0.51) scores were higher than for the EQ-5D (R^2 ^0.20 - 0.35), suggesting that the SF-6D mapping model explained more of the variance in observed scores (Table 3). The predicted mean (SD) baseline EQ-5D in BROSG patients did not differ from observed values (EQ-5D: observed 0.59 (0.22) vs. predicted 0.59 (0.19), p = 0.494). The predicted mean EQ-5D values were significantly higher than the observed values in STIVEA, (observed 0.47 (0.31) vs. predicted 0.53 (0.25), p < 0.001) and those in the BSRBR (observed 0.40 (0.33) vs. predicted 0.44 (0.26), p < 0.001). The variance around all predicted utility values was consistently lower than that around observed values i.e. the predicted values were falsely precise.

**Table 3:** Comparison of baseline observed and predicted utility scores

		Observed	Predicted		Difference (Observed-Predicted)	
	n	Mean (SD)	Mean (SD)	**R**^2^	Mean (95% CI)	95% reference range
EQ-5D						
STIVEA	224	0.47 (0.30)	0.53 (0.25)	0.35	0.06 (0.02, 0.09)	-0.44 to 0.56
BROSG	453	0.59 (0.22)	0.59 (0.19)	0.20	0.01 (-0.01, 0.03)	-0.42 to 0.44
BSRBR	795	0.40 (0.33)	0.44 (0.26)	0.35	0.04 (0.02, 0.06)	-0.49 to 0.57
SF-6D						
STIVEA	224	0.57 (0.13)	0.67 (0.07)	0.45	0.10 (0.09, 0.11)	-0.09 to 0.29
BROSG	453	0.63 (0.13)	0.68 (0.07)	0.34	0.05 (0.04, 0.05)	-0.16 to 0.25
BSRBR	795	0.53 (0.11)	0.63 (0.07)	0.51	0.09 (0.09, 0.10)	-0.06 to 0.25

Abbreviations: BROSG = British Rheumatoid Outcome Study Group, BSRBR = British Society for Rheumatology Biologics Register, EQ-5D = EuroQol-5D, SF-6D = Short Form-6D, STIVEA = Steroids In Very Early Arthritis

Predicted SF-6D scores were consistently higher than observed scores (Table 3) across all cohorts. The predicted mean baseline SF-6D for BROSG patients was a small over-estimate (observed 0.63 (0.13) vs. predicted 0.68 (0.07), p < 0.001). However, predicted mean SF-6D values were considerably higher than observed values in STIVEA (observed 0.57 (0.13) vs. predicted 0.67 (0.07), p < 0.001) or the BSRBR (observed 0.53 (0.11) vs. predicted 0.65 (0.06), p < 0.001).

### Longitudinal analysis

Complete EQ-5D, SF-6D and HAQ details were available for 1283 patients at baseline and the final follow-up assessment. The HAQ scores of patients in the STIVEA trial (1 year mean change -0.38 (SD 0.66)) and BSRBR study (6 month mean change -0.27 (SD 0.87)) improved over the follow-up period (results not provided in tables). The mean HAQ score of patients in the BROSG trial deteriorated (3 year mean change 0.16 (SD 0.47)). There was moderate correlation of change in HAQ with change in EQ-5D in STIVEA (r = 0.58) and with change in SF-6D in STIVEA (r = 0.68) and BSRBR (r = 0.53). Lower correlations of change in HAQ and EQ-5D were observed in BROSG (r = 0.33) and BSRBR (r = 0.42) and with the SF-6D in BROSG (0.31) (results not provided in tables).

The R^2 ^values for the relationship between change in observed and predicted SF-6D scores (R^2 ^0.11 - 0.46) were once more higher than for the EQ-5D (R^2 ^0.08 - 0.22) (Table 4). Change in predicted values of the EQ-5D (mean difference 0.00, 95% CI -0.02, 0.03) and SF-6D (mean difference -0.00, 95% CI -0.01, 0.01) corresponded very well with observed change in patients from the BROSG study, a group with established disease (Table 4). The change in predicted and observed EQ-5D scores was also very similar in patients receiving anti-TNF therapy (mean difference -0.01, 95% CI -0.04, 0.01).

**Table 4:** Change in observed and predicted utility scores

		Observed	Predicted		Difference (Observed-Predicted)	
Study, follow-up	n	Mean (SD)	Mean (SD)	**R**^2^	Mean (95% CI)	95% reference range
EQ-5D						
STIVEA, 1-year	159	0.20 (0.31)	0.12 (0.24)	0.22	-0.07 (-0.12, -0.03)	-0.50 to 0.64
BROSG, 3-year	375	-0.06 (0.24)	-0.06 (0.24)	0.08	-0.00 (-0.02, 0.02)	-0.50 to 0.50
BSRBR, 6-month	749	0.08 (0.33)	0.07 (0.25)	0.19	-0.01 (-0.04, 0.01)	-0.60 to 0.63
SF-6D						
STIVEA, 1-year	159	0.13 (0.16)	0.04 (0.07)	0.46	-0.09 (-0.11, -0.07)	-0.14 to 0.33
BROSG, 3-year	375	-0.02 (0.11)	-0.02 (0.05)	0.11	-0.00 (-0.01, 0.01)	-0.21 to 0.21
BSRBR, 6-month	749	0.05 (0.12)	0.02 (0.06)	0.33	-0.03 (-0.03, -0.02)	-0.16 to 0.21

Abbreviations: BROSG = British Rheumatoid Outcome Study Group, BSRBR = British Society for Rheumatology Biologics Register, EQ-5D = EuroQol-5D, SF-6D = Short Form-6D, STIVEA = Steroids In Very Early Arthritis

Predicted EQ-5D scores significantly underestimated change in patients with early arthritis (mean difference -0.07, 95% CI -0.12, -0.03). The mean change in predicted SF-6D scores was less than half that in observed values in patients with early arthritis (SF-6D: observed 0.13 (SD 0.16) vs. predicted 0.04 (SD 0.07), p < 0.001) and severe RA (SF-6D: observed 0.05 (SD 0.12) vs. predicted 0.02 (SD 0.06), p < 0.001). There was no significant difference in change using predicted and observed SF-6D values in the BRSOG trial.

## Discussion

We found that, using the method of Bansback *et al*. [8], the validity of estimating utility scores from the HAQ varies according to disease activity and duration. Predicted values overestimated values cross-sectionally and underestimated change in patients with active arthritis, particularly those with very early disease. These differences were clinically significant; the difference between observed and predicted SF-6D exceeded the estimated minimum important difference (MID) for this measure (0.03-0.04)[15] for all cross-sectional baseline estimates and for change over 6 months in the very early disease group. Predicted SF-6D values overestimated baseline values and underestimated improvement in patients with active disease by approximately 60-70%. Similarly, the difference between observed and predicted values of the EQ-5D at baseline and for change over time in the very early disease patients were in the range of previous estimates of the MID for this measure (0.05-0.13)[15]. Estimating change in EQ-5D and SF-6D scores in patients with more stable established disease was more accurate. Overall, EQ-5D scores predicted from the HAQ were more accurate than SF-6D scores predicted from the HAQ.

On the basis of our results, it seems likely that evaluations of QALYs derived by mapping from the HAQ may provide conservative estimates of cost-effectiveness of treatments. In other words, the number of QALYs gained by the treatment may be underestimated and so the cost per QALY will appear higher than it actually is. Conservative cost-effective ratios might therefore incorrectly impact on the decisions by organizations such as NICE in the UK[2], increasing the likelihood of truly cost effective treatments being rejected if predicted/mapped utility values were used. NICE states that a single consistent measurement and valuation of health-related quality of life, preferably the EQ-5D, is required to assess the effectiveness of an intervention [16]. However, NICE recognises that the EQ-5D is not always collected, and in these circumstances suggests that methods may be used to estimate EQ-5D utility values by mapping. A recent study estimating EQ-5D values from the Western Ontario and McMaster Universities Osteoarthritis (WOMAC) index also reported that QALY gains and cost per QALY estimated using mapped and actual EQ-5D values were very different. Our study emphasizes the need, in future studies, to incorporate preference based instruments such as the EQ-5D or SF-36 or SF-12 which allow the calculation of the SF-6D [5,17], and supports the similar recommendations made by Barton et al [18].

During the analysis for this study we attempted to develop a consistent model to estimate the EQ-5D and SF-6D from the HAQ using the three cohorts of patients reflecting a range of arthritis states and severity of disease. We performed closed-test comparisons for alternative fractional polynomial model specifications but found no improvement on the model specified by Bansback *et al*. [8]. We also attempted to use the additional covariates of age, sex, disease duration and DAS28 score, but remained unable to develop a prediction model which explained the difference in the relationship between the HAQ and EQ-5D/SF-6D within our three cohorts.

As expected [19] we found that predicted utility scores have smaller variance than observed values. This is because mapped values lack the within person variance found in observed values. Therefore, in addition to mapped utility values resulting in an inflated cost per QALY estimate, the probability of a treatment being cost-effective at a specified level of willingness to pay (e.g. £20-30 k in the UK), which is driven by uncertainty around the cost and effect parameter estimates, will also be overestimated. One way to solve this particular issue may be to use multiple imputation of utility values, rather than a single imputation as performed here. Furthermore, the ability to predict the SF-6D and EQ-5D from the HAQ is complicated by the weighting of items in the EQ-5D and SF-6D profiles into the preference-based utility values. Therefore the contribution of each of the domains to the eventual health states is complex and compounded by potential change over time in each of the domains. The ability to predict the domain scores of the EQ-5D and SF-6D, possibly using multiple predictors, which can then be converted to an overall summary score through the respective algorithms may improve the accuracy of prediction.

Although Scott *et al*., reporting that the EQ-5D and HAQ were unrelated in measuring change (r = 0.08) [20], we found correlations of change scores to be considerably higher (EQ-5D and HAQ: 0.33 - 0.58). The data in this study suggest that, in certain situations, mapping from the HAQ to the EQ-5D or SF-6D may be acceptable. The results suggest that the mean EQ-5D for a group of patients predicted from the HAQ is better estimate than the mean SF-6D predicted from the HAQ than the SF-6D when using the methods of Bansback, *et al*. [8]. In previous studies in RA using direct measurement, the EQ-5D has been shown to correlate more strongly with measures of functional disability and damage than the SF-6D [21-23]. Although the moderate to high correlations of the HAQ and SF-6D and higher R^2 ^for the relationship between observed and predicted SF-6D scores, suggesting the potential for mapping between the HAQ and SF-6D, the systematic differences between observed and predicted SF-6D scores are worrying since they suggest that the mapping function investigated in this study introduces bias. The poorer performance of predicted utility values in patients with more active disease, where pain and fatigue may play a greater role, counsels against mapping utility scores for measures of functional disability alone in this context. This might also explain the poorer performance of the predicted SF-6D, a measure appears to have a better descriptive ability for patients with less severe disease [21], compared with the EQ-5D in this study, which contrasts with the lower reported root mean square error for predicted versus observed SF-6D values than EQ-5D values reported by Bansback *et al*. [8].

A recent study by Amjadi, *et al*[10] evaluated the validity of SF-6D scores predicted by the methods described by Bansback, *et al*. [8] finding that predicted SF-6D scores were valid in terms of the type of tests usually applied in the validation an outcome measure, namely (construct validity: correlation with other patient reported and clinical outcome measures, and discrimination patients with differing severity of disease defined as tertiles of a range of VAS scales) and responsiveness to change assessed against clinical anchors (in this case change on a range of 100 mm visual analogue scales ≥ 10 mm). However the assessment did not included head-to-head assessment of the predicted measure compared to the observed measure, and was conducted in a single patient group. This might mean that although the predicted measure may detect clinically important change in a patient group, whether this is an over- or under-estimate of the 'real' change that would have been detected by collection of the actual measure can not be assessed. For example, with data presented in this study we might conclude that the predicted SF-6D was able detect a clinically important mean change of 0.04 (i.e. >MID[15]) in the STIVEA patients, however comparison with observed SF-6D data (mean change 0.13) reveals that this is a considerable underestimate.

## Conclusions

In conclusion, we suggest that estimation of utility values from the HAQ in studies of patients with inflammatory arthritis should be undertaken with caution, particularly in those with active disease. On the basis of the difference between observed and predicted scores, mapping of the EQ-5D from the HAQ appeared to be more valid than mapping the HAQ to the SF-6D, particularly in patients with established stable disease. Further research is required to determine whether EQ-5D and SF-6D values in patients with more active disease, can be predicted using extra covariates (as well as the HAQ). However estimating utility scores is demonstrably inferior to collecting the utility measures as part of a study. Our findings support the recommendations of OMERACT, and more recently Barton et al [18] to include at least one measure of HRQoL, specifically one which allows the estimation of utilities, in all relevant clinical studies.

## Abbreviations

BROSG: British Rheumatoid Outcome Study Group; BSRBR: British Society for Rheumatology Biologics Register; DAS28: Disease Activity Score based on 28 swollen and tender joint counts; EQ-5D: EuroQol-5D; ESR: Erythrocyte Sedimentation Rate; HAQ: Health Assessment Questionnaire; HRQoL: Health-Related Quality of Life; IQR: Interquartile Range; NICE: National Institute for Health and Clinical Excellence; OMERACT: Outcome Measures in Rheumatology; QALYs: Quality-Adjusted Life Years; RA: Rheumatoid Arthritis; RCT: Randomised Controlled Trial; SF-36 - Short Form 36-Item Health Survey; SF-6D: Short Form-6D; STIVEA: Steroids In Very Early Arthritis; TNFα: Tumour Necrosis Factor Alpha; WOMAC: Western Ontario and McMaster Universities Osteoarthritis.

## Competing interests

The authors declare that they have no competing interests.

## Authors' contributions

MH participated in the design of the study and performed the statistical analysis and interpretation of data, and drafted the manuscript; ML participated in the design of the study and the statistical analysis and was involved in revising the manuscript critically for important intellectual content; SV made substantial contributions to the acquisition of the data, was involved in drafting and revising the manuscript critically for important intellectual content; KW made substantial contributions to the acquisition of the data, was involved in drafting and revising the manuscript critically for important intellectual content; NB contributed to the analysis and interpretation of data, and was involved in drafting and revising the manuscript critically for important intellectual content; DS made substantial contributions to conception and design, and interpretation of data, and was involved in drafting the manuscript or revising it critically for important intellectual content. All authors read and approved the final manuscript.
